# Health-Related Quality of Life in Spanish Women with Eating Disorders

**DOI:** 10.3390/nu13020403

**Published:** 2021-01-27

**Authors:** Isabel Panea-Pizarro, José M. Moran, Jesús Lavado-García, Luis Beato-Fernández, Ana Teresa Domínguez-Martin, Sara Huerta-González, Andre Novo, Juan D. Pedrera-Zamorano, Fidel López-Espuela

**Affiliations:** 1 Mental Health Department, Hospital General Universitario, 13001 Ciudad Real, Spain; isabelpanea.pizarro@hotmail.com (I.P.-P.); lbeato@sescam.jccm.es (L.B.-F.); 2 Metabolic Bone Diseases Research Group, Nursing Department, Nursing and Occupational Therapy College, University of Extremadura, Avd. Universidad s/n, 10003 Cáceres, Spain; jmmorang@unex.es (J.M.M.); jpedrera@unex.es (J.D.P.-Z.); fidellopez@unex.es (F.L.-E.); 3 Nursing Department, Hospital Universitario de Caceres, 10003 Cáceres, Spain; anat_dm@hotmail.com; 4 Nursing College, Universidad Veracruzana, Región Poza Rica, Tuxpan 92870, Mexico; sahuerta@uv.mx; 5 Nursing Department, Instituto Politecnico de Bragança, 5300-252 Bragança, Portugal; andrenovo@gmail.com

**Keywords:** health-related quality of life, eating disorders, anorexia nervosa, bulimia nervosa, binge eating disorder, other specified feeding or eating disorder

## Abstract

People with eating disorders show impaired health-related quality of life (HRQoL). We aimed to investigate the relative role of physical and mental factors and stage of change as possible predictors of HRQoL in a group of Spanish women (*n* = 124) with eating disorders. For this purpose, initial and follow-up data were obtained after 6 months from patients attending an outpatient treatment unit for eating disorders. The determinants of the physical and mental domains of the Medical Outcomes Survey Short-form Health Survey (SF-36) questionnaire were investigated in the total sample and separately based on the eating disorder diagnosis by multiple linear regression. Lower scores in the physical component of the SF-36 questionnaire were associated with the presence of a higher body mass index (BMI) at follow-up as well as a higher score in the “action” component of the Attitudes towards Change in Eating Disorders Questionnaire (ACTA). Conversely, a higher index in the EuroQoL-5D overall quality of life questionnaire (EQ-5D) and the presence of obsessive compulsive disorder were associated with a higher score in the physical dimension. The instrument used demonstrated the ability to assess changes associated with the physical component of these patients over the period studied, and the analysis provided more information and specific data on different aspects of HRQoL, thus allowing a more detailed analysis of the information.

## 1. Introduction

Health-related quality of life (HR-QoL) refers to the set of effects on physical, mental and social health as well as the subjective perception of health as evaluated and indicated by the patients themselves [1,2]. This concept has been used in the past to quantitate the burden related to physical disease and to evaluate the results of certain treatments. The World Health Organization Quality of Life (WHOQOL) Group provided one of the most frequently used definitions: “‘individuals’ perceptions of their position in life in the context of the culture and value systems in which they live and in relation to their goals, expectations, standards and concerns’’ [3]. People with eating disorders show significant deterioration in the physical, psychological and social dimensions [4,5], having established that that this disorder has a negative effect on the HRQoL of these patients [6,7,8,9,10,11,12] [for a review see: [13]]. An eating disorder is characterized by abnormal eating behavior that results in either insufficient or excessive food intake as well as accompanying feelings of distress or concern about body weight or shape. It sometimes occurs in combination with compensatory behavior, to the detriment of the person’s physical health [6]. The Fifth Diagnostic and Statistical Manual of Mental Disorders (DSM-5) covered several inadequacies observed in previous editions by broadening the diagnostic definitions of anorexia nervosa and bulimia nervosa and increasing the number of diagnostic categories, adding binge eating disorder as a separate diagnostic entity, and adding a new category, other specified eating or feeding disorders [14].

Even subclinical episodes (subjective bulimic episodes) have been shown to be predictive of HRQoL impairment in samples of patients with eating disorders (anorexia nervosa, bulimia nervosa, binge eating disorder and other specified feeding or eating disorder) [15]. The precise identification of the different roles mental and physical disorders may play as determinants of HRQoL in patients with eating disorders may be medically relevant. In fact, we can assume that HRQoL may improve as treatment is established or, on the contrary, that the improvement in HRQoL is less relevant, or only temporary, if a low HRQoL prior to the establishment of treatment was the result of mental factors not completely overcome or corrected by treatment. Considering the relevance of eating disorders in today’s society, their high prevalence [16], and the associated morbidity and mortality [17,18], it is important to more precisely determine their impact on HRQoL.

HRQoL determination in patients with eating disorders through both generic and specific questionnaires is consolidated in the scientific literature [19]. Thus, HRQoL determinations could be described as generic or disease-specific [20]. These generic HRQoL questionnaires are intended to measure HRQoL when applied to all (or the majority of) people without regard to a given group or disease state. The Medical Outcomes Survey Short-form Health Survey (SF-36) [2] is a generic health-related quality of life assessment self-report questionnaire. This generic questionnaire is widely used when assessing HRQoL in eating disorders, and one large U.S. study reported high internal consistency reliability scores for all subscales (>0.8) [21]. This questionnaire has also allowed detection of changes in the physical and mental dimensions of patients with eating disorders in longitudinal follow-ups of medium range (6 months) [22]. Other questionnaires that analyze HRQoL from a general perspective are also beginning to be used in populations with eating disorders, such as the EuroQoL-5D overall quality of life questionnaire (EQ-5D) [23]. The strength of this instrument lies in the fact that it is a comprehensive and validated assessment tool that identifies the health status preferences of the patients and is easy to implement. Nevertheless, EQ-5D has not been widely tested among people with eating disorders or those who report disordered eating behaviors [24].

The lack of motivation for treatment is one of the most surprising features of people with eating disorders. The Prochaska and DiClemente Trans-Theoretical Model (TTM) is a conceptual model that represents different stages of motivation [25]. Five different stages of motivation were initially proposed, in which a person could be: in the “precontemplation stage,” with no awareness or intention to change the problematic behavior, in the “contemplation stage,” where there is awareness of the problematic behavior and the intention to change it but no appropriate action is being taken, in the “preparation stage,” where there is an intention to change the problematic behavior and a subsequent compromise to do so within the near future, or in the “action stage,” where there is an ongoing effort to change the problematic behavior. During the “maintenance stage,” the problem behavior has been changed, and now there is an attempt to sustain these changes [25,26]. Attitudinal and behavioral changes through these five stages do not flow in a linear progression to the next stage; rather, they spiral up through the different stages. Thus, there is a consecutive progression from one stage to another, but there is always the possibility of returning to a previous stage. Patients with eating disorders show different levels of motivation to change different features of the eating disorder. These patients may be highly motivated to stop eating compulsively but may not be prepared to consider changing their restrictive dietary behavior. Determining a person’s stage of change is particularly relevant for disorders with a behavioral dimension (e.g., eating disorders) [27].

Considering the potential influence and consequences of eating disorders on women’s health, particularly in the psychosocial setting, it is important to explore whether eating disorders may be associated with HRQoL as a measure of health outcome in women. The aim of our study was, therefore, to investigate the relative role of physical and mental factors and stage of change as possible descriptors of HRQoL in a group of Spanish women with eating disorders. For this purpose, initial and follow-up data were obtained after 6 months in patients attending an outpatient treatment unit for eating disorders.

## 2. Materials and Methods

This was a prospective observational study conducted between February 2018 and November 2018. Participants were selected intentionally. Clinical and sociodemographic characteristics of the sample at baseline have been reported previously [28]. The sample consisted of 124 Spanish women aged 27.3 (10.5) with similar education levels and residing in the same area. The participants exhibited symptoms of the following medically diagnosed eating disorders: anorexia nervosa, bulimia nervosa, other specified feeding or eating disorder and binge eating disorder. All of the women were undergoing treatment in an outpatient unit for eating disorders in the General University Hospital of Ciudad Real (Spain). Each patient was asked to complete the EuroQol EQ-5D, the SF-36, the Eating Attitudes Test-26 (EAT-26) and the Attitudes towards Change in Eating Disorders Questionnaire (ACTA) questionnaires at baseline and at 6 months. The number of participants who answered all the questionnaires at baseline was *n* = 124, while it was *n* = 120 at the 6-month follow-up (3.2% dropout rate). Additionally, sociodemographic and clinical information was collected from the participants at the beginning and at the follow-up of the 6 months, including age, weight, body mass index (BMI), type of eating disorder, smoking habit, hospitalization and associated time, marital status, educational level, mental comorbidities as well as the presence of autolytic attempts.

### 2.1. Survey Short-Form Health Survey

The SF-36 [2] is a 36-item measure of everyday functioning ability. The SF-36 measures the generic quality of life from the patient’s perspective. It has 8 separate component scores that can be combined into 2 summative components: the physical and mental components. The eight dimensions evaluated include physical functionality, physical role, physical pain, general health, vitality, social functioning, mental health and emotional role. In this scale, two summary measures were used: the summary of the physical component of health (PCS) and summary of the mental health component (MCS). High scores indicate greater impairment of HRQoL, and low scores indicate less deterioration in quality of life. Summary results have proven to be robust and reliable measures of HRQoL [2] and are valuable in the evaluation of health impairments of patients with eating disorders [7,9].

### 2.2. Eating Attitudes Test-26

The EAT-26 questionnaire is a validated survey that consists of 3 scales: diet (includes fattening food avoidance behaviors and concern about thinness), bulimia and concern about food (bulimic behaviors: binge eating and vomiting as well as thoughts about food) and oral control (self-control over intake and pressure from others to gain weight). Each question has a possible answer between 6 (never, rarely, sometimes, often, almost always, always) scored according to the Likert scale 000123. Only item 25 is scored in the opposite way, by scoring 0, 0, 0, 1, 2, 3 (“never” = 3). The optimal cut-off point varies between 10–20 according to different authors; in general, a score above the cut-off point of 20 implies the need for further research. Although its primary use is as a screening test, it has been described as a predictive test because it is considered sensitive to therapeutic changes or changes in the symptomatology of eating disorder patients [29].

### 2.3. Evaluation of Results in Patient-Centered Health: Quality of Life

All patients in the study were asked to complete the EuroQoL-5D overall quality of life questionnaire (EQ-5D) [23]. The EQ-5D is a generic instrument comprising a visual analogue scale (VAS) of self-rated general health and 5 dimensions (mobility, self-care, daily activities, pain/discomfort, and anxiety/depression). There are five possible answer options for each question, ranging from “no problem” to “serious problem.” Both scales have been validated for the Spanish general population. Scores on the VAS can range from 0 (worse state) to 100 (best state). An interviewee marks their subjectively perceived health condition on the thermometer scale. Scores on the 5 dimensions can be expressed as an overall summary index (EQ-5D index) or as the percentage of patients who indicate some kind of problem on each of the dimensions. The EQ-5D score (HRQoL dimensions) has values ranging from 0 to 1 (0—worst health condition, 1—best health condition).

### 2.4. Attitudes towards Change in Eating Disorders Questionnaire (ACTA)

ACTA is a 59-point questionnaire adapted from the idea of staging an algorithm in patients with eating disorders [30,31]. The participants answered on a Likert scale ranging from 0 to 4 (from “no/never” to “yes/never”), varying according to the degree to which they were affected by various change activities. Each scale was scored separately (precontemplation, reflection, preparation, action and maintenance), with the level of change predominantly scoring the highest. The survey also provides a scale for estimating deterioration or relapse. This is very reliable, with alpha coefficients from 0–74 to 0.90 for each of the six modified subcategories [31]. The subscales are clearly correlated with one another and with the questionnaires for the measurement of eating psychopathology. After the analysis of the factors, they correspond strongly to the six subcategories, which supports their constructed validity.

### 2.5. Ethics Consideration

Procedures were established in concordance with the Declaration of Helsinki and approved by the Ethics and Clinical Research Committee of Ciudad Real (Spain) (ref. 2017C/123). All patients signed a written informed consent form to participate in the study.

### 2.6. Statistical Analysis

The data were analyzed using SPSS software version 23.0 (SPSS, Chicago, IL, USA). Frequencies, mean and standard deviation or median values and interquartile range (IQR) were used to describe the baseline and follow-up characteristics of the patients. A Kruskal–Wallis test followed by post hoc Dunn’s test was used to compare continuous variables between eating disorder groups. Longitudinal changes in quantitative variables were analyzed using the Wilcoxon test. To compare the percentage of categorical variables in different groups, the Chi square test was used. The non-parametric marginal homogeneity test/Stuart-Maxwell test was used to test paired measurements of the EQ-5D test. To determine the relationships between continuous variables, multiple linear regression was used. Scores of the two summary measures (physical component summary scale and mental component summary scale) of the SF-36 were entered as dependent variables. Age, BMI, marital status (single, married), education level (primary school, high school, university), mental comorbidities (depression, anxiety, OCD, eating disorder (anorexia nervosa, bulimia nervosa, other specified feeding or eating disorder, binge eating disorder), EQ-5D-3L (index and VAS), ACTA dimensions (precontemplation, contemplation, preparation, action, maintenance, relapse) and EAT-26 scores were entered as independent variables. Predictors were selected at a significance level of <0.05. Stepwise multiple linear regression was performed with the predictors entered or removed from the model according to the following criteria: probability-of-F-to-enter ≤0.050, probability-of-F-to-remove ≥0.100. Statistical significance was set at *p* < 0.05.

## 3. Results

### 3.1. Clinical and Sociodemographic Data

The reported eating disorder composition of the sample was anorexia nervosa (47.6%), bulimia nervosa (28.2%), other specified feeding or eating disorder (15.3%) and binge eating disorder (8.9%). The mean age of the sample was 27.3 (10.5) years. The majority (82.1%) had never been married, and 32.3% had a university education. The mean BMI was 22.18 (8.38). There were no differences in BMI or weight between baseline and at the 6-month follow-up (*p* = 0.710 and *p* = 0.730, respectively, Table 1). The mental disorders participants reported undergoing treatment for were: depression (41.2%), anxiety (45.9%) and obsessive compulsive disorder (OCD) (12.9%), with minor changes at the 6-month follow-up (Table 1). Overall, 79% of the sample reported having had autolytic attempts.

### 3.2. Health-Related Quality of Life (HRQoL) by Eating Disorder

Patients were categorized according to their diagnosed eating disorder and then compared by the HRQoL standardized scores of the SF-36 subscales. As shown in Table 2, statistically significant differences (*p* = 0.014) were observed at baseline on the physical functioning subscale, with significantly lower scores in patients with binge eating disorder (35 (15) *versus* anorexia nervosa (80 (50)) (*p* = 0.002) and bulimia nervosa (80 (60)) (*p* = 0.005). After the 6-month follow-up, significant differences were also registered in the physical functioning subscale, with the binge eating disorder group again scoring lower (45 (55)) (*p* = 0.01). Conversely, in the body pain subscale after 6 months of follow-up, statistically significant differences were observed, with the highest score registered in the binge eating disorder group (40 (40) (*p* = 0.01) versus anorexia nervosa (10 (40) (*p* = 0.01) and bulimia nervosa (0 (40)) (*p* = 0.02). Significant improvements in the physical functioning subscale were observed at follow-up for the anorexia nervosa (*p* < 0.001), bulimia nervosa (*p* = 0.009), and other specified feeding or eating disorder (*p* = 0.007) groups as well as for the general health subscale (*p* = 0.038) in women with anorexia nervosa. No changes in the different subscales were observed for the group of women diagnosed with binge eating disorder (Table 2).

We then compared the summary scores based on the diagnosis of eating disorder (Table 3). Statistically significant differences between the study groups were reported both at baseline and at the 6-month follow-up. At baseline and related to the physical domain, patients diagnosed with anorexia nervosa reported higher scores than the binge eating disorder group (*p* = 0.045). At 6 months of follow-up, differences were reported between binge eating disorder versus other specified feeding or eating disorders (*p* = 0.027), binge eating disorder versus anorexia nervosa (*p* < 0.0001) and anorexia nervosa versus bulimia nervosa (*p* = 0.011). In the mental domain, on the contrary, the highest score was obtained in the binge eating disorder group (41.86 (8.5)) with respect to anorexia nervosa (*p* = 0.018). Significant differences were also reported between anorexia nervosa and bulimia nervosa (*p* = 0.012). Similar results were obtained at the 6-month follow-up, with the binge eating disorder group reporting the highest score in the mental domain (41.46 (6.89)) (*p* = 0.006). In the physical domain after 6 months of follow-up, significant improvements were observed in the scores of the anorexia nervosa (*p* = 0.004), bulimia nervosa (*p* = 0.039) and binge eating disorder (*p* = 0.043) groups, with no noticeable changes in the physical domain for the other specified feeding or eating disorder groups.

**Figure 1:**
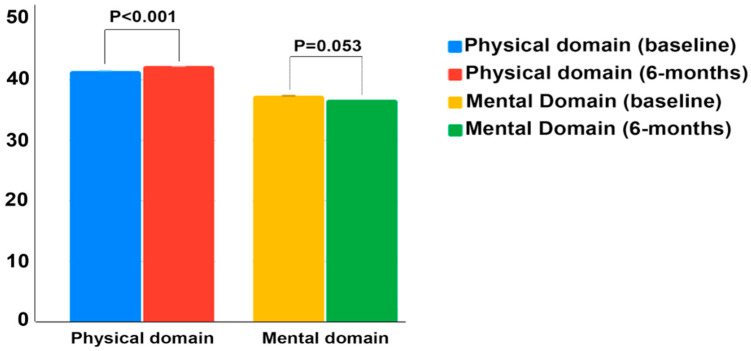
Distribution of SF-36 physical and mental summary scores of patients.

With respect to mental domain, no changes were observed within groups after 6 months of follow-up. In the entire sample, regarding the physical and mental domains, a significant improvement in the physical domain was observed after 6 months of follow-up (41.92(8.52) versus 42.46 (8.43); *p* < 0.001)), while no change was observed in the summary scores of the mental domain (36.75 (8.43) versus 36.33 (8.03); *p* = 0.053)) (Figure 1).

Total SF-36 physical and mental score distributions in the entire sample were recorded. Comparisons between baseline and at the 6-month follow-up by Wilcoxon signed rank test.

We continued to evaluate the HRQoL of study participants by administering the EQ-5D questionnaire. Differences between the study groups were observed, with the exceptions of pain (*p* = 0.483) and anxiety (*p* = 0.122) at baseline and self-care (*p* = 0.460), activity (*p* = 0.079) and pain (*p* = 0.088) at 6 months (Table 4). The results of the particular dimensions of the questionnaire are presented in Table 4, separated into the four different groups of pathologies studied. We observed changes along the temporal follow-up for the “mobility” dimension in the bulimia nervosa group (*p* = 0.025) and “activity” for the binge eating disorder group (*p* = 0.025), in both cases reflecting a substantial improvement of the patients’ situation (Table 4).

No other changes related to time follow-up were observed. However, the indexes derived from the application of the EQ-5D questionnaire did not reflect any change either in the total sample or when analyzing them by study group. No statistically significant differences were observed either at the baseline level between groups (*p* = 0.103 for EQ-VAS and *p* = 0.095 for EQ-Index) or after the 6-month follow-up (*p* = 0.146 for EQ-VAS and *p* = 0.143 for EQ-Index). There were also no changes observed in the indexes as a function of time within the study groups (*p* > 0.05 for all the comparisons) (Table 5).

### 3.3. Attitudes towards Change in Eating Disorders

The median scores for the total sample for the phases of precontemplation (21 (7)), contemplation (23.6 (4.1)), action (25 (9)) and relapse (22.8 (12.1)) did not change significantly over the 6-month follow-up (*p* > 0.05 for all matches) (Table 6). There was a significant decrease in the preparation subscale (263 (7.2) versus 25 (5); *p* = 0.001) as well as an improvement in the maintenance subscale (10.5 (8)) versus 15.0 (9); *p* = 0.013). Regarding the scores of all stages, a Kruskal–Wallis test showed no significant differences between the diagnostic subgroups (anorexia, bulimia, other specified feeding or eating disorders and binge eating disorder), with the sole exception of “maintenance” at the beginning of the study (*p* = 0.006). The scores of the anorexia nervosa group were significantly lower than those observed in the bulimia nervosa group (*p* = 0.009) and the binge eating disorder group (*p* = 0.005). No other statistically significant differences were noted either at baseline or in the follow-up after 6 months.

### 3.4. Eating Attitudes Test-26

Within the total study sample, a decrease in the total score in the EAT-26 test was observed throughout the study (52 (30) versus 47 (30); *p* = 0.022). In the prospective follow-up, this significant decrease was only observed within the group of women with anorexia (59 (11) versus 54 (31); *p* = 0.002); no other statistically significant differences were reported as a function of time. The intergroup comparison at the baseline stage showed statistically significant differences (*p* < 0.001) with the anorexia nervosa group, with a significantly higher score on the EAT-26 test than that observed in the anorexia nervosa (*p* < 0.001) and binge eating disorder (*p* < 0.001) groups. No statistically significant differences were observed between groups at the 6-month follow-up according to eating disorder diagnosis (Table 7).

### 3.5. Determinants of HRQoL

The determinants of the physical and mental domains of the SF-36 questionnaire were investigated in the total sample and separately according to eating disorder diagnosis through a step-by-step multiple linear regression analysis (Table 8). In the total sample, lower scores in the physical component of the SF-36 questionnaire were associated with the presence of a higher BMI at follow-up as well as a higher score in the “action” component of the ACTA questionnaire at follow-up. Conversely, the presence of a higher index in the EQ-5D questionnaire and the presence of OCT were associated with a higher score in the physical dimension of the questionnaire. In any case, the proposed model only explained a moderate fraction of the associated variability for the physical component. The model responds to a variability of 41.3% in the total sample (Table 8). Related to the mental dimension of the SF-36 questionnaire and regarding the total sample, a higher score in the EAT-26 questionnaire at the onset of the study was associated with a lower score in the mental dimension (Table 8). Conversely, a higher score in the VAS scale of the EQ-5D questionnaire was associated with a higher score in the mental dimension of the SF-36. The model proposed in our study could only respond to a minimum fraction of the associated variability (22%) for the mental component. Subsequently, the main determinants of the mental and physical components of the SF-36 questionnaire were analyzed according to the eating disorder diagnosis. The results obtained are shown in Table 8. For the physical dimension of the SF-36 questionnaire, a higher score at the beginning of the study on the EQ-5D index acted as a positive predictor in women with anorexia nervosa. No negative predictors were identified, allowing for the proposed model to account for up to 31% of the variability associated with the physical dimension in this study group. No positive or negative determinants were identified for women with bulimia nervosa or other specified feeding or eating disorders for the physical dimension of the SF-36 questionnaire (Table 8). The model proposed for women with binge eating disorder regarding the physical component of the SF-36 questionnaire could explain up to 73% of the associated variability by including only the VAS score associated with the EQ-5D at the baseline of the study (positive determinant) (Table 8). Regarding the mental dimension in the different groups studied, a higher score at the beginning of the study in the EAT-26 questionnaire was associated with a lower score in anorexic women. The proposed model would explain, however, a minor fraction of the associated variability (27%) in this group of women. In the group of women with bulimia nervosa, the EQ-5D (VAS) at the beginning of the study was a positive determinant, providing a model that could explain up to 41% of the variability associated with the mental dimension of SF-36. There were no predictors of the mental dimension in women with other specified feeding or eating disorders. Finally, in the group of women with binge eating disorder, age was the sole (positive) determinant of the score associated with the mental dimension of SF-36 through a model that would explain up to 80% of the variability associated with that dimension (Table 8).

Results of a stepwise multiple linear regression analysis are represented. Scores of the two summary measures (physical component summary scale and mental component summary scale) of the SF-36 were entered as dependent variables. Age, BMI, marital status (single, married), education level (primary school, high school, university), mental comorbidities (depression, anxiety, OCT), eating disorder (anorexia nervosa, bulimia nervosa, other specified feeding or eating disorder, binge eating disorder), EQ-5D-3L (index and VAS), ACTA dimensions (precontemplation, contemplation, preparation, action, maintenance, relapse) and EAT26 scores were entered as independent variables. Predictors were selected at a significance level of <0.05. B-coefficient, standard error and *p*-value are indicated.

## 4. Discussion

We observed statistically significant improvements in the physical domain of HRQoL in patients with eating disorders after 6 months of multidisciplinary treatment using the SF-36 questionnaire. Overall, patients with anorexia nervosa, bulimia nervosa and binge eating disorder experienced the greatest advances in the physical dimension but no changes in the mental dimension. Patients with other specified feeding or eating disorders experienced no significant improvements in either the physical or mental domain. Our study confirms previous findings showing that the generic HRQoL tool, the SF-36, is useful for monitoring the impairment of health in patients with eating disorders. Patients with anorexia nervosa, bulimia nervosa, and other specified feeding or eating disorders showed functional improvements in their daily activities, which were reflected in significant increases in the physical role in SF-36. These results are consistent with those previously reported in the Spanish population, given that no clinically relevant differences were detected between the different domains of SF-36 according to the eating disorder diagnosis [32]. However, some authors have argued that use of generic questionnaires, such as the SF-36, may produce erroneous HRQoL data in patients with eating disorders [7]. The researchers suggested that the SF-36 may not be sensitive to screening for emotional stress in these patients; for example, the increased physical activity associated with improved quality of life in the SF-36 may be a sign of severity in anorexia nervosa rather than improvement [23].

Although similar to our results in that certain improvements were observed, our study may have underestimated some favorable results that could not have been detected due to the moderate follow-up time applied. Follow-ups of up to 8 years have shown significant improvements in HRQoL of patients with eating disorders [33]. These long follow-up studies have also reported no clinically relevant differences in HRQoL between different eating disorder groups. Furthermore, they have also shown that the recovery of the physical domain and the quality of life associated therewith is much more constant in the literature [6]. In contrast, women with anorexia nervosa who achieved physical recovery after five years of treatment required up to five more years to achieve recovery within the mental domain [34]. This suggests that, after the physical recovery of patients, it is necessary to continue the follow-up since they can continue to maintain a lasting effect on their HRQoL over time. Nonetheless, long-term HRQoL studies in patients with eating disorders are limited and would be more appropriate for addressing questions about whether patients eventually reach the level of the healthy population. Short-term and medium-term designs, in contrast, would allow for investigating whether the generally multidisciplinary treatments implemented improve HRQoL.

In our study, the physical component of the SF-36 questionnaire at the end of the follow-up was negatively affected by the BMI and by the quality of life at the beginning of the study. This result is consistent with that observed in other studies, in which multivariate models indicated that BMI acts as a negative predictor of decreases in HRQoL [24]. The BMI was already mostly under control in our study and as a consequence of the treatment to which patients were being subjected. The result observed may reflect the fact that patients with higher BMI could be experiencing more serious eating disorders, and these would then be associated with a more severe HRQoL impairment. No precise data were available on the evolution of the patients’ BMI prior to the study; thus, it was not possible to fully explore the implications of this result. Although this result was observed in the total sample, in the group analysis, BMI was not a relevant predictor of either the physical or mental component. Although we are cautious about this result due to the small sample size derived from the subgroup analysis, it agrees with other previous reports in patients with binge eating disorder whose BMI was not significantly associated with HRQoL, suggesting that the relationship between the diagnosis of binge eating disorder and HRQoL is not fully explained by weight status [11,35,36].

Regarding the HRQoL determined with the EQ-5D instrument, the moderate results obtained in the current study are consistent with other results obtained after two years of follow-up in patients with anorexia nervosa, where differences could be observed relative to EQ-VAS but not the EQ-5D index scores [37]. This may reflect a lack of sensitivity of the 5 dimensions composing the EQ-5D to accurately assess the day-to-day difficulties faced by patients with anorexia nervosa. Part of the explanation could be that the participants usually score highly on dimensions related to mobility and self-care because some activities, such as excessive or compulsive physical exercise (reflecting their anorexia nervosa symptoms), may cause them to perceive themselves as mobile and self-care oriented [37]. This could also explain why the presence of OCT was detected as a positive predictive factor of the physical domain of the HRQoL.

The stage of change in eating disorders is a strong predictor of treatment outcome [38]. Significant changes in the entire sample were reported in our study regarding the stages of change. The literature is scarce about studies that have established relationships between the stage of change and HRQoL. A decrease in follow-up with respect to “preparation” and an increase in “maintenance” were observed. With respect to HRQoL, increases in action scores imply decreases in the physical component. With the exception of “maintenance,” all patients were in the same phase regardless of diagnosis; thus, the stage of change did not relate to diagnostic category, confirming previous findings [31,39]. Higher “action” scores have been described as predictors of changes in BMI one year after treatment, reflecting weight gain in anorexic patients and weight loss in bulimic patients [31], which does not coincide with our results regarding the physical component of the HRQoL. Certainly, our findings present moderate-term follow-up results, and longer-term follow-ups as reported in the literature may reflect other types of evolution with respect to the stage of change experienced by the patients. It is possible that, because patients use both counterconditioning and stimulus control procedures to actively change their behavior during the action stage, this may affect their HRQoL in some way. Nevertheless, due to the uniqueness of this result and the intrinsic limitations of our study design, no clinically relevant conclusions may be derived from this result.

In patients with anorexia nervosa, impairments, especially in the mental domains of SF-36, have been associated with higher scores in the EAT-26, in line with what was observed in our study for the anorexia nervosa group and for the total sample. The relationship is partially attributable to the fact that the treatment of eating disorders tends to stabilize the physical aspects by pursuing a progressive and gradual improvement of the clinical conditions of the patients. The mental components are more difficult to modify because they are rooted in the structure of the individual’s personality and because it is a psychiatric disorder of known complexity that, as indicated above, persists even years after physical recovery [40].

We recognize several limitations in our study. We only analyzed data from a group of women so our study cannot provide insights into the possible influence of gender on the HRQoL of patients diagnosed with eating disorders. Self-reported scales, rather than data collection through interviews, may limit the value of the findings of our study. The sample size was limited; although it was almost entirely maintained during the prospective follow-up, small sample sizes were observed when performing the cluster analysis. This can have an impact on the ability to detect effect sizes since we could be incurring a type II error due to a lack of statistical power. Furthermore, although the design has the advantage of being a longitudinal design, it did not allow us to establish cause–effect relationships in order to identify the factors that influence possible changes or their absence in the HRQoL. A major limitation of our study was the use of generic instruments to determine quality of life instead of dedicated ones to measure quality of life in patients with eating disorders. These questionnaires are more suitable for the specific characteristics of the disorders being investigated. Patients generally feel that the questions are directly related to their own pathology, thus improving the reliability of the instrument. Nevertheless, disease-specific quality of life questionnaires would not allow comparisons between diagnostic groups. Another important limitation that we recognize is that the data were collected from two sampling points. Data from more than two measurements and more separated in time (i.e., several follow-up periods) can more clearly describe the importance, effect, and interactions of possible risk factors for poorer HRQoL. There may have been mixed or conflicting results due to either individual differences or changes within individuals in relation to the standards values or conceptualization of the HRQoL [33,41,42]. Finally, Axis I disorders can contribute to affecting the HRQoL. We recognize an important limitation in our work in that no information was collected on other possible comorbidities such as Axis I disorders as well as treatment with psychotropic medications that could influence the results of the study.

Therefore, through the use of generic instruments to assess HRQoL, an effective determination of HRQoL has been achieved in a sample of Spanish women with eating disorders before and after six months of treatment and follow-up. The instrument used demonstrated the ability to assess changes associated with the physical component of these patients over the period studied, and the analysis provided more information and specific data on different aspects of HRQoL, thus allowing a more detailed analysis of the information. Future research should address longer follow-up periods and explore direct comparisons between specific instruments for determining HRQoL in patients with eating disorders and generic instruments to better outline any changes in HRQoL.

## Figures and Tables

**Table 1:** Clinical and sociodemographic data from patients with eating disorders at baseline and at 6-months of follow-up (*n* = 124).

Variable	Mean (SD)/*n* (%)	Median	IQR	*p*-Value (Baseline vs. 6-Months) ^a^
Age (years)	27.3 (10.5)	25	17	
Weight (kg)	28.4 (23.1)	50.9	15.5	0.710
Weight (6-months) (kg)	28.5 (24.4)	50.0	17.8	
Height (meters)	1.62 (0.06)	1.62	0.09	
Body mass index (BMI) (kg/m^2^)	22.18 (8.38)	19.19	6.04	0.730
BMI (6-months) (kg/m^2^)	22.19 (8.79)	19.05	6.37	
Smoking ^#^				
Yes	68 (54.80%)			
No	56 (45.20%)			
Eating disorder ^#^				
Anorexia nervosa	59 (47.6%)			
Bulimia nervosa	35 (28.2%)			
Other specified feeding or eating disorder	19 (15.3%)			
Binge eating disorder	11 (8.9%)			
Years since the eating disorder diagnosis (years)	10.3 (7.9)	8.0	12.5	
Years of treatment in the eating disorders unit (years)	8 (6.5)	6.0	10	
Ever hospitalized ^#^				
Yes	64 (51.6%)			
No	60 (48.4%)			
Marital status ^#^				
Married	22 (17.9%)			
Single	102 (82.1%)			
Education level ^#^				
Low (primary school)	5 (4%)			
High school	79 (63.7%)			
University	40 (32.3%)			
Mental health (comorbidities)				
Depression	35 (41.2%)			
Anxiety	39 (45.9%)			
Obsessive-compulsive disorder	11 (12.9%)			
Mental health (comorbidities) (6-months)				
Depression	35 (40.7%)			
Anxiety	38 (44.2%)			
Obsessive-compulsive disorder	13 (15.1%)			
Autolytic attempt ^#^				
Yes	99 (79.8%)			
No	25 (20.20%)			

^#^ No change after 6 months of follow-up. ^a^ Wilcoxon signed-rank test.

**Table 2:** Distribution of the Medical Outcomes Survey Short-form Health Survey (SF-36) standardized scores by eating disorder group.

SF-36 Subscales	Anorexia Nervosa (*n* = 59)	Bulimia Nervosa (*n* = 35)	Other Specified Feeding or Eating Disorder (*n* = 19)	Binge Eating Disorder (*n* = 11)	
	Median (IQR)	Median (IQR)	Median (IQR)	Median (IQR)	*p*-Value *
Baseline (*n* = 124)					
Physical functioning	80 (50)	80 (60)	60 (45)	35 (15)	0.014 ^1^
Physical role	75 (100)	75 (100)	25 (100)	0 (75)	0.113
Bodily pain	5 (20)	0 (40)	20 (40)	20 (20)	0.114
General health	57 (5.75)	56.25 (12)	56.25 (8)	57 (7)	0.224
Vitality	65 (10)	65 (10)	65 (15)	60 (15)	0.696
Social functioning	50 (25)	50 (25)	50 (12.5)	50 (37.5)	0.917
Emotional role	0 (33.33)	33.33 (66.67)	0 (33.33)	0 (66.67)	0.2
Mental health	60 (8)	64 (12)	60 (16)	64 (12)	0.623
6-month follow-up (*n* = 120)					
Physical functioning	90 (30) (*p* < 0.001))	64 (12) (*p* = 0.009)	80 (35) (*p* = 0.007)	45 (55)	0.01 ^2^
Physical role	75 (100)	90 (60)	25 (100)	0 (75)	0.113
Bodily pain	10 (40)	0 (40)	20 (40)	40 (40)	0.01 ^3^
General health	57 (6.25) (*p* = 0.038)	56.25 (10)	57 (8)	60 (12)	0.08
Vitality	65 (10)	65 (10)	65 (15)	60 (15)	0.696
Social functioning	50 (12.5)	50 (25)	50 (25)	50 (25)	0.484
Emotional role	0 (33.33)	33.33 (66.67)	0 (33.33)	0 (66.67)	0.2
Mental health	60 (8)	64 (12)	60 (16)	64 (12)	0.623

* Between subtypes of eating disorders at baseline or 6-months of follow-up (Kruskal–Wallis test). ^1^ Post hoc analysis by Dunn’s test: Binge eating disorder vs. Anorexia nervosa (*p* = 0.002) and Bulimia nervosa (*p* = 0.005) were statistically significant. Other pairwise comparisons were not statistically significant (*p* > 0.05). ^2^ Post hoc analysis by Dunn’s test: Binge eating disorder vs. Anorexia nervosa (*p* = 0.001) and Bulimia nervosa (*p* = 0.003) were statistically significant. Binge eating disorder vs. Other specified feeding or eating disorder (*p* = 0.046) was statistically significant. Other pairwise comparisons were not statistically significant (*p* > 0.05). ^3^ Post hoc analysis by Dunn’s test: Binge eating disorder vs. Anorexia nervosa (*p* = 0.01) and Bulimia nervosa (*p* = 0.02) were statistically significant. Bulimia nervosa vs. Anorexia nervosa (*p* = 0.012) was statistically significant. Other pairwise comparisons were not statistically significant (*p* > 0.05). Longitudinal basline-6 months follow-up comparisons by Wilcoxon signed rank test.

**Table 3:** Total SF-36 physical and mental scores by eating disorder.

SF-36 Summary Scores	Anorexia Nervosa (*n* = 59)	Bulimia Nervosa (*n* = 35)	Other Specified Feeding or Eating Disorder (*n* = 19)	Binge Eating Disorder (*n* = 11)	
	Median (IQR)	Median (IQR)	Median (IQR)	Median (IQR)	*p*-Value *
Baseline					
Physical domain	43.44 (8.69)	40.6 (6.38)	41.96 (9.79)	33.94 (8.32)	0.045 ^1^
Mental Domain	35.32 (7.48)	39.51 (9.51)	36.12 (10.72)	41.86 (8.5)	0.02 ^2^
6-month follow-up					
Physical domain	44.56 (8.3) (*p* = 0.004)	41.02 (6.76) (*p* = 0.039)	41.77 (9.22)	35.92 (6.99) (*p* = 0.043)	0.001 ^3^
Mental Domain	35.04 (7.55)	37.93 (8.36)	37.37 (8.38)	41.46 (6.89)	0.006 ^4^

* Comparisons between eating disorders by the Kruskal-Wallis test. ^1^ Post hoc analysis by Dunn’s test: Binge eating disorder vs. Anorexia nervosa (*p* = 0.008). Other pairwise comparisons were not statistically significant (*p* > 0.05). ^2^ Post hoc analysis by Dunn’s test. Binge eating disorder vs. Anorexia nervosa (*p* = 0.018); Bulimia nervosa vs. Anorexia nervosa (*p* = 0.012). Other pairwise comparisons were not statistically significant (*p* > 0.05). ^3^ Post hoc analysis by Dunn’s test. Binge eating disorder vs. Other specified feeding or eating disorder (*p* = 0.027); Binge eating disorder vs. Anorexia nervosa (*p* < 0.0001); Anorexia nervosa vs. Bulimia nervosa (*p* = 0.011). Other pairwise comparisons were not statistically significant (*p* > 0.05). ^4^ Post hoc analysis by Dunn’s test. Anorexia nervosa vs. Bulimia nervosa (*p* = 0.005); Anorexia nervosa vs. Binge eating disorder (*p* = 0.006). Other pairwise comparisons were not statistically significant (*p* > 0.05). Longitudinal basline-6 months follow-up comparisons by Wilcoxon signed rank test. *p* < 0.05 is marked as bold.

**Table 4:** Dimensions of the EuroQoL-5D overall quality of life questionnaire (EQ-5D-3L) test at baseline (*n* = 124) and at 6-months of follow-up (*n* = 120) in the studied sample.

Items		Total Sample *N* (%)	Anorexia Nervosa (*n* = 59) *N* (%)	Bulimia Nervosa (*n* = 35) *N* (%)	Other Specified Feeding or Eating Disorder (*n* = 19) *N* (%)	Binge Eating Disorder (*n* = 11) *N* (%)	*p*-Value ^a^
Mobility (baseline)	I have no problems walking about	107 (86.3%)	57 (96.6%)	28 (80%)	17 (89.5%)	5 (45.50%)	<0.001
	I have some problems walking about	17 (13.7%)	2 (3.4%)	7 (7%)	2 (10.5%)	6 (54.50%)	
	I am confined to bed	0 (0%)	0 (0.0%)	0 (0%)	0 (0%)	0 (0.00%)	
Mobility (6-months)	I have no problems walking about	112 (93.3%)	56 (98.2%	32 (94.1%)	17 (94.4%)	7 (63.6%)	0.001
	I have some problems walking about	7 (5.8%)	0 (0%)	2 (5.9%)	1 (5.6%)	4 (36.4%)	
	I am confined to bed	1 (0.8%)	1 (1.8%)	0 (0%)	0 (0%)	0 (0%)	
*p*-value ^b^		0.059	1	0.025	0.564	0.317	
Self-care (baseline)	I have no problems with self-care	120 (96.8%)	58 (98.3%)	35 (100%)	19 (100%)	8 (72.70%)	<0.001
	I have some problems with self-care	4 (3.20%)	1 (1.7%)	0 (0%)	0 (0%)	3 (27.30%)	
	I am unable to wash or dress myself	0 (0%)	0 (0%)	0 (0%)	0 (0%)	0 (0.00%)	
Self-care (6-months)	I have no problems with self-care	116 (96.7%)	55 (96.5%)	34 (100%)	17 (94.4%)	10 (90.9%)	0.460
	I have some problems with self-care	4 (3.3%)	3 (3.5%)	0 (0%)	1 (5.6%)	1 (9.1%)	
	I am unable to wash or dress myself	0 (0%)	0 (0%)	0 (0%)	0 (0%)	0 (0%)	
*p*-value ^b^		1	0.564	N/A	0.317	0.317	
Activity (baseline)	I have no problems with performing my usual activities	95 (76.6%)	52 (88.1%)	25 (71.4%)	15 (78.9%)	3 (27.3%)	0.001
	I have some problems with performing my usual activities	25 (20.2%)	6 (10.2%)	9 (25.7%)	4 (21.1%)	6 (54.6%)	
	I am unable to perform my usual activities	4 (3.2%)	1 (1.7%)	1 (2.9%)	0 (0%)	2 (18.2%)	
Activity (6-months)	I have no problems with performing my usual activities	96 (80%)	47 (82.5%)	30 (88.2%)	13 (72.2%)	6 (54.5%)	0.079
	I have some problems with performing my usual activities	24 (20%)	10 (17.5%)	4 (11.8%)	5 (27.8%)	5 (45.5%)	
	I am unable to perform my usual activities	0 (0%)	0 (0%)	0 (0%)	0 (0%)	0 (%)	
*p*-value ^b^		0.206	0.439	0.071	0.665	0.025	
Pain (baseline)	I have no pain or discomfort	84 (67.7%)	45 (76.3%)	22 (62.9%)	12 (63.2%)	5 (45.50%)	0.483
	I have moderate pain or discomfort	33 (26.6%)	12 (20.3%)	10 (28.6%)	6 (31.6%)	5 (45.50%)	
	I have extreme pain or discomfort	7 (5.6%)	2 (3.4%)	3 (9.6%)	1 (5.3%)	1 (9.10%)	
Pain (6-months)	I have no pain or discomfort	76 (63.3%)	42 (73.7%)	17 (50.0%)	12 (66.7%)	5 (45.5%)	0.088
	I have moderate pain or discomfort	39 (32.5%)	12 (21.1%)	17 (50.0%)	5 (27.8%)	5 (45.5%)	
	I have extreme pain or discomfort	5 (4.2%)	3 (5.3%)	0 (0%)	1 (5.6%)	1 (9.1%)	
*p*-value ^b^		0.522	0.353	0.827	0.705	1	
Anxiety (baseline)	I am not anxious or depressed	44 (35.5%)	16 (27.1%)	19 (54.3%)	5 (26.3%)	4 (36.40%)	0.122
	I am moderately anxious or depressed	55 (44.4%)	32 (54.2%)	11 (31.4%)	8 (42.1%)	4 (36.40%)	
	I am extremely anxious or depressed	25 (20.2%)	11 (18.6%)	5 (14.3%)	6 (31.6%)	3 (27.30%)	
Anxiety (6-months)	I am not anxious or depressed	49 (40.8%)	25 (43.9%)	13 (38.2%)	6 (33.3%)	5 (45.5%)	0.047
	I am moderately anxious or depressed	41 (34.2%)	21 (36.8%)	15 (44.1%)	2 (11.1%)	3 (27.3%)	
	I am extremely anxious or depressed	30 (25%)	11 (19.3%)	6 (17.6%)	10 (55.6%)	3 (27.3%)	
*p*-value ^b^		0.927	0.327	0.223	0.637	0.808	

^a^ Chi-square test. ^b^ Marginal homogeneity test/Stuart–Maxwell test.

**Table 5:** Health-related quality of life according to the EQ-5D-3L scores in Spanish women with eating disorders.

	Total Sample	Anorexia Nervosa (*n* = 59)	Bulimia Nervosa (*n* = 35)	Other Specified Feeding or Eating Disorder (*n* = 19)	Binge Eating Disorder (*n* = 11)	*p*-Value ^a^
	Median (IQR)	Median (IQR)	Median (IQR)	Median (IQR)	Median (IQR)	
EQ-VAS (baseline)	79.89 (35.46)	79.86 (26.1)	79.86 (35.46)	79.86 (32.27)	64.54 (46.75)	0.103
EQ-VAS (6-months follow-up)	79.44 (46.45)	79.86 (26.1)	79.02 (11.47)	53.55 (31.82)	79.02 (66.89)	0.146
*p*-value ^b^	0.560	0.941	0.351	0.264	0.314	
EQ-Index (baseline)	0.914 (0.29)	0.914 (0.175)	0.914 (0.29)	0.914 (0.462)	0.71 (0.639)	0.095
EQ-Index (6-months follow-up)	0.901 (0.469)	0.914 (0.175)	0.887 (0.16)	0.541 (0.444)	0.887 (0.725)	0.143
*p*-value ^b^	0.599	0.786	0.799	0.223	0.374	

^a^ Kruskal–Wallis test. ^b^ Wilcoxon Signed Rank Test.

**Table 6:** Median scores on Attitudes towards Change in Eating Disorders Questionnaire (ACTA) subscales at baseline (*n* = 124) and 6-months of follow-up (*n* = 120) in women with eating disorders.

Subscale	Total Sample Mean (SD)	Anorexia Nervosa (*n* = 59) Median (IQR)	Bulimia Nervosa (*n* = 35) Median (IQR)	Other Specified Feeding or Eating Disorder (*n* = 19) Median (IQR)	“Binge Eating Disorder (*n* = 11)” Median (IQR)	*p*-Value ^a^
Precontemplation (baseline)	21 (7)	22 (6)	20 (7)	21 (5)	19 (11)	0.590
Precontemplation (6-months follow-up)	20.5 (7)	19.5 (8.5)	22 (6)	19 (7.5)	20 (5)	
*p*-value ^b^	0.384	0.251	0.269	0.271	0.953	
Contemplation (baseline)	23.6 (4.1)	23.6 (3.63)	22.7 (6.3)	24.5 (3.6)	24.5 (10.9)	0.492
Contemplation (6-months follow-up)	24 (6)	24 (4.5)	24 (4)	19.5 (9.5)	21 (7)	
*p*-value ^b^	0.217	0.877	0.976	0.133	0.407	
Preparation (baseline)	26.3 (7.2)	27.2 (6.3)	25.4 (6.3)	26.3 (7.2)	27.2 (7.2)	0.888
Preparation (6-months follow-up)	25 (5)	25 (5)	24 (4)	23 (75)	24 (6)	
*p*-value ^b^	0.001	0.122	0.021	0.066	0.374	
Action (baseline)	25 (9)	24 (9)	26 (9)	23 (7)	26 (8)	0.680
Action (6-months follow-up)	24 (5.5)	24.5 (6.5)	23 (5)	25 (5)	23 (7)	
*p*-value ^b^	0.122	0.092	0.122	0.209	0.593	
Maintenance (baseline)	10.5 (8)	9 (7)	11 (8)	12 (8)	13 (11)	0.006 ^1^
Maintenance (follow-up)	15 (9)	15 (9.5)	14 (8)	16 (14)	14 (7)	
*p*-value ^b^	0.013	0.119	0.148	0.127	0.953	
Relapse (baseline)	22.8 (12.1)	22.85 (10)	20 (15.7)	27.1 (11.4)	22.8 (24.2)	0.190
Relapse (follow-up)	23 (7)	23 (4)	23 (7)	21.5 (12)	20 (8)	
*p*-value ^b^	0.314	0.794	0.988	0.013	0.635	

^a^ Kruskal–Wallis test. ^b^ Wilcoxon Signed Rank test. ^1^ Post hoc analysis by Dunn’s test: Anorexia nervosa vs. Bulimia nervosa (*p* = 0.009) or Binge eating disorder (*p* = 0.005) were statistically significant. Other pairwise comparisons were not statistically significant (*p* > 0.05).

**Table 7:** Eating Attitudes Test-26 (EAT-26) test scores in the total sample and by eating disorder. Baseline data (*n* = 124) and follow-up after 6 months (*n* = 120).

	Total Sample Median (IQR)	Anorexia Nervosa (*n* = 59) Median (IQR)	Median (IQR)	Other Specified Feeding or Eating Disorder (*n* = 19) Median (IQR)	“Binge Eating Disorder (*n* = 11)” Median (IQR)	*p*-Value ^a^
EAT score (baseline)	52 (30)	59 (11)	37 (39)	50 (29)	17 (39)	*p* < 0.001 ^1^
EAT score (6-month follow-up)	47 (30)	54 (31)	38 (27)	51 (21)	29 (35)	0.088
*p*-value ^b^	0.022	0.002	0.650	0.513	0.260	

^a^ Kruskal–Wallis test. ^b^ Wilcoxon Signed Rank test. ^1^ Post hoc analysis by Dunn’s test: Anorexia nervosa vs. Bulimia nervosa (*p* < 0.001) or Binge eating disorder (*p* < 0.001) were statistically significant. Other pairwise comparisons were not statistically significant (*p* > 0.05).

**Table 8:** Determinants of health-related quality of life (HRQoL) in 124 Spanish women with eating disorders enrolled in a 6-month prospective observational study.

	Total Sample		Anorexia Nervosa (*n* = 59)		Bulimia Nervosa (*n* = 35)		Eating Disorder Not Otherwise Specified (*n* = 19)	Binge Eating Disorder (*n* = 11)	
	Explained Variance	Significant Predictors	Explained Variance	Significant Predictors	Significant Predictors		Significant Predictors	Explained Variance	Significant Predictors
Physical domain	R2 = 0.413	EQ-5D index (baseline): B = 7.139 (1.981); *p* = 0.001	R2 = 0.315	EQ-5D index (baseline): B = 15.231 (3.798): *p* < 0.001	No significant predictors detected.		No significant predictors detected.	R2 = 0.738	EQ-5D (VAS) (baseline): B = 14.266 (3.214): *p* = 0.003
		BMI (follow-up): B = −0.213 (0.062): *p* = 0.001							
		ACTA (action) (follow-up): B = −0.247 (0.077): *p* = 0.02							
		OCT (baseline): B = 4.619 (1.581): *p* = 0.005							
Mental domain	R2 = 0.220	EAT26 (baseline): B = −0.131 (0.033): *p* < 0.001	R2 = 0.270	EAT26 (baseline): B = −0.199 (0.055): *p* = 0.001	R2 = 0.412	EQ-5D (VAS) (baseline): B = 15.905 (4.149): *p* = 0.001	No significant predictors detected.	R2 = 0.805	Age: B = 0.521 (0.097): *p* = 0.001
		EQ-5D (VAS) (baseline): B = 7.233 (2.647): *p* = 0.008							

## Data Availability

The data presented in this study are available on request from Dr. Fidel Lopez-Espuela.

## References

[1] Heithoff K.A., Lohr K., Institute of Medicine (US) Division of Health Care (1990). Assessing Health-Related Quality of Life Outcomes.

[2] Ware J.E., Sherbourne C.D. (1992). The MOS 36-item short-form health survey (SF-36). I. Conceptual framework and item selection. Med. Care.

[3] The WHOQOL Group (1998). Development of the World Health Organization WHOQOL-BREF quality of life assessment. Psychol. Med..

[4] Becker A.E., Grinspoon S.K., Klibanski A., Herzog D.B. (1999). Eating Disorders. N. Engl. J. Med..

[5] Hsu L.K.G. (1990). Eating Disorders.

[6] De la Rie S.M., Noordenbos G., van Furth E.F. (2005). Quality of life and eating disorders. Qual. Life Res..

[7] Doll H.A., Petersen S.E., Stewart-Brown S.L. (2005). Eating disorders and emotional and physical well-being: Associations between student self-reports of eating disorders and quality of life as measured by the SF-36. Qual. Life Res..

[8] González-Pinto A., Inmaculada F., Cristina R., de Corres Blanca F., Sonsoles E., Fernando R., Purificacion L. (2004). Purging behaviors and comorbidity as predictive factors of quality of life in anorexia nervosa. Int. J. Eat. Disord..

[9] Hay P. (2003). Quality of life and bulimic eating disorder behaviors: Findings from a community-based sample. Int. J. Eat. Disord..

[10] Pollack L.O., McCune A.M., Mandal K., Lundgren J.D. (2015). Quantitative and Qualitative Analysis of the Quality of Life of Individuals With Eating Disorders. Prim. Care Companion CNS Disord..

[11] Singleton C., Kenny T.E., Hallett D., Carter J.C. (2019). Depression Partially Mediates the Association Between Binge Eating Disorder and Health-Related Quality of Life. Front. Psychol..

[12] Ágh T., Kovács G., Supina D., Pawaskar M., Herman B.K., Vokó Z., Sheehan D.V. (2016). A systematic review of the health-related quality of life and economic burdens of anorexia nervosa, bulimia nervosa, and binge eating disorder. Eat. Weight Disord..

[13] Jenkins P.E., Hoste R.R., Meyer C., Blissett J.M. (2011). Eating disorders and quality of life: A review of the literature. Clin. Psychol. Rev..

[14] American Psychiatric Association (2013). Diagnostic and Statistical Manual of Mental Disorders (DSM-5^®^).

[15] Latner J.D., Vallance J.K., Buckett G. (2008). Health-related quality of life in women with eating disorders: Association with subjective and objective binge eating. J. Clin. Psychol. Med. Settings.

[16] Peláez-Fernández M.A., Raich R., Labrador F. (2010). Eating disorders in Spain: Revision of empirical epidemiological studies. Mex. J. Eat. Disord..

[17] Smith A.R., Ortiz S.N., Forrest L.N., Velkoff E.A., Dodd D.R. (2018). Which Comes First? An Examination of Associations and Shared Risk Factors for Eating Disorders and Suicidality. Curr. Psychiatry Rep..

[18] Thomas J.J., Vartanian L.R., Brownell K.D. (2009). The relationship between eating disorder not otherwise specified (EDNOS) and officially recognized eating disorders: Meta-analysis and implications for DSM. Psychol. Bull..

[19] Engel S.G., Adair C.E., Las Hayas C., Abraham S. (2009). Health-related quality of life and eating disorders: A review and update. Int. J. Eat. Disord..

[20] Fayers P., Machin D. (2007). Quality of Life: The Assessment, Analysis and Interpretation of Patient-Reported Outcomes.

[21] Leung S.F., Ma J.L., Russell J. (2013). Enhancing quality of life in people with disordered eating using an online self-help programme. J. Eat. Disord..

[22] Vancampfort D., Probst M., Adriaens A., Pieters G., De Hert M., Stubbs B., Soundy A., Vanderlinden J. (2014). Changes in physical activity, physical fitness, self-perception and quality of life following a 6-month physical activity counseling and cognitive behavioral therapy program in outpatients with binge eating disorder. Psychiatry Res..

[23] Badia X., Roset M., Herdman M., Kind P. (2001). A comparison of United Kingdom and Spanish general population time trade-off values for EQ-5D health states. Med. Decis. Mak..

[24] Hart L.M., Gordon A.R., Sarda V., Calzo J.P., Sonneville K.R., Samnaliev M., Austin S.B. (2020). The association of disordered eating with health-related quality of life in U.S. young adults and effect modification by gender. Qual. Life Res..

[25] Prochaska J.O., DiClemente C.C. (1983). Stages and processes of self-change of smoking: Toward an integrative model of change. J. Consult. Clin. Psychol..

[26] Junne F., Ziser K., Mander J., Martus P., Denzer C., Reinehr T., Wabitsch M., Wiegand S., Renner T., Giel K.E. (2016). Development and psychometric validation of the ‘Parent Perspective University of Rhode Island Change Assessment-Short’ (PURICA-S) Questionnaire for the application in parents of children with overweight and obesity. BMJ Open.

[27] Hasler G., Delsignore A., Milos G., Buddeberg C., Schnyder U. (2004). Application of Prochaska’s transtheoretical model of change to patients with eating disorders. J. Psychosom. Res..

[28] Panea-Pizarro I., López-Espuela F., Martos-Sánchez A., Domínguez-Martín A.T., Beato-Fernández L., Moran-García J.M. (2020). Internet addiction and Facebook addiction in Spanish women with eating disorders. Arch. Psychiatr. Nurs..

[29] Loria Kohen V., Gómez Candela C., Lourenço Nogueira T., Pérez Torres A., Castillo Rabaneda R., Villarino Marin M., Bermejo López L., Zurita L. (2009). Evaluation of the utility of a Nutrition Education Program with Eating Disorders. Nutr. Hosp..

[30] Beato Fernández L., Rodríguez Cano T. (2003). Attitudes towards change in eating disorders (ACTA). Development and psychometric properties. Actas Esp. Psiquiatr..

[31] Rodríguez-Cano T., Beato-Fernández L. (2005). Attitudes towards change and treatment outcome in eating disorders. Eat. Weight Disord..

[32] Padierna A., Quintana J.M., Arostegui I., Gonzalez N., Horcajo M.J. (2000). The health-related quality of life in eating disorders. Qual. Life Res..

[33] Pohjolainen V., Koponen S., Räsänen P., Roine R.P., Sintonen H., Karlsson H. (2016). Long-term health-related quality of life in eating disorders. Qual. Life Res..

[34] Keski-Rahkonen A., Hoek H.W., Susser E.S., Linna M.S., Sihvola E., Raevuori A., Bulik C.M., Kaprio J., Rissanen A. (2007). Epidemiology and course of anorexia nervosa in the community. Am. J. Psychiatry.

[35] Masheb R.M., Grilo C.M. (2004). Quality of life in patients with binge eating disorder. Eat. Weight Disord..

[36] Grenon R., Tasca G.A., Cwinn E., Coyle D., Sumner A., Gick M., Bissada H. (2010). Depressive symptoms are associated with medication use and lower health-related quality of life in overweight women with binge eating disorder. Womens Health Issues.

[37] Strand M., Bulik C.M., Gustafsson S.A., von Hausswolff-Juhlin Y., Welch E. (2020). Self-admission to inpatient treatment in anorexia nervosa: Impact on healthcare utilization, eating disorder morbidity, and quality of life. Int. J. Eat. Disord..

[38] Iyar M.M., Cox D.W., Kealy D., Srikameswaran S., Geller J. (2019). Is stage of change enough? Confidence as a predictor of outcome in inpatient treatment for eating disorders. Int. J. Eat. Disord..

[39] Gusella J., Butler G., Nichols L., Bird D. (2003). A brief questionnaire to assess readiness to change in adolescents with eating disorders: Its applications to group therapy. Eur. Eat. Disord. Rev..

[40] Oliveira-Cardoso É.A., Coimbra A.C., Santos M.A. (2018). Quality of life of patients with anorexia and Bulimia Nervosa. Psicol. Teoria Pesqui..

[41] Schwartz C.E., Andresen E.M., Nosek M.A., Krahn G.L., RRTC Expert Panel on Health Status Measurement (2007). Response shift theory: Important implications for measuring quality of life in people with disability. Arch. Phys. Med. Rehabil..

[42] Sprangers M.A.G., Schwartz C.E. (1999). Integrating response shift into health-related quality of life research: A theoretical model. Soc. Sci. Med..

